# Quantitative analysis of the impact of infectious disease physicians on patients in the emergency department fast-track parenteral antibiotics program

**DOI:** 10.1186/s12879-024-09305-0

**Published:** 2024-07-01

**Authors:** C. McSweeney, T. Steiner, J. M. Grant

**Affiliations:** 1 Jim Pattison Pavilion, 899 W 12th Ave, V5Z 1M9 Vancouver, BC Canada; 2 Resident Physician University of British Columbia, Vancouver General Hospital, 596 Davis Drive, L3Y 2P9 Vancouver, Newmarket, BC, Ontario Canada; 3 Division of Infectious Diseases, Department of Medicine, Faculty of Medicine, University of British Columbia, Vancouver General Hospital, Vancouver, BC Canada; 4 Division of Infectious Diseases, Division of Medical Microbiology, University of British Columbia, Vancouver General Hospital, Vancouver, BC Canada

**Keywords:** OPAT, IV antibiotics, Emergency medicine, Parenteral antibiotics, Cellulitis

## Abstract

**Introduction:**

The outpatient parenteral antibiotic therapy (OPAT) program of Vancouver General Hospital (VGH) was supervised by emergency physicians (EPs) until 2017 when infectious disease (ID) physicians began assisting in management. We designed a retrospective study to determine whether ID involvement led to improved outcomes.

**Methods:**

This study analyzes the impact of ID involvement by comparing the mean days patients spent on OPAT with ID involvement versus EPs alone through a retrospective chart review. Secondary research objectives were to compare patient care decisions, e.g., antibiotic choice, tests ordered, and final diagnosis.

**Results:**

There was no difference between the mean number of days on OPAT between physician types. Compared to historic patterns, patients seen in OPAT after increased ID consultation spent an average of 0.5 fewer days in the program. However, when grouped by the first day of ID assessment, the average total days in OPAT was closely aligned with the day of first ID assessment, implying that ID frequently discharged patients close to initial assessment. Patients seen by ID were less likely to return within one month of discharge compared to those not seen by ID. Secondary findings include ID physicians prescribing a greater range of antibiotics, providing more varied final diagnoses, prescribing antibiotics less frequently, as well as ordering more cultures, diagnostic imaging and specialist consults.

**Discussion:**

The findings of this study support the hypothesis that ID involvement in OPAT programs leads to changes in care that may have beneficial outcomes for patients and the healthcare system.

**Supplementary Information:**

The online version contains supplementary material available at 10.1186/s12879-024-09305-0.

## Introduction

Outpatient parenteral antibiotic treatment (OPAT) programs have become a widespread treatment modality that reduces financial burden on the healthcare system [1]. OPAT allows ambulatory treatment of patients with infections that necessitate intravenous (IV) antibiotics, but do not require in-hospital care. Infections that are commonly treated with OPAT include: septic arthritis, osteomyelitis, infective endocarditis, cellulitis, and many others [2, 3]. OPAT comes in many forms in Canada, including at-home treatment (home IV), OPAT clinics, and emergency department (ED) visits [3].

Prior to 2021, VGH (Vancouver General Hospital)– a large, quaternary-care academic medical centre in Vancouver, Canada– had an OPAT run by the ED, without routinely involving Infectious disease (ID) specialists. Patients were admitted to the OPAT program on their initial ED visit and returned (typically once daily) to receive parenteral antibiotics under the supervision of the emergency physician (EP) on shift, with ID consultation at the discretion of the EP– although this was rarely done. Beginning in the fall of 2017, ID specialists began supervision of the OPAT program. With this change, EPs were encouraged to refer patients to a dedicated ID physician on or before their fifth day in the OPAT. While this involvement was done in consultation with the EPs, it remains unknown whether it improved quality of care or patient outcomes.

Moderate-to-severe cellulitis was the leading infectious diagnosis for patients in the OPAT program. We retrospectively compared the management of cellulitis in OPAT patients treated by EPs alone and those treated by ID physicians at least once. We also compared patients treated before and after the introduction of ID physician involvement to determine if this comparison would support the permanent inclusion of ID physicians throughout OPAT programs.

## Methods

The OPAT program at VGH was previously run by EPs. Starting on November 1st 2017 an ID physician was assigned to the OPAT program each week from Monday to Friday from 9 am to noon. EPs were encouraged to refer patients to ID physicians after the 5th day of treatment, unless the EP felt ID physician involvement was not indicated. Patients were seen by EPs on duty if they came into OPAT outside of ID supervised hours. Limited hours and lack of presence on weekends and holidays meant that many patients were still managed by EPs alone.

A retrospective chart review of patients with cellulitis in the VGH ED OPAT program from November 1st 2016 to November 1st 2018 was performed. These dates capture one year before through one year after the increased involvement of ID physicians. We performed a contemporaneous comparison of patients seen by only EPs vs. those seen at least once by ID physicians. Since no patients were seen only by ID physicians the comparison was made between those seen by ID physicians at least once and those never seen by ID physicians during their time in OPAT.

Charts were identified using ED diagnostic codes. The inclusion criteria were patients coded as “IV antibiotics” with an initial diagnosis listed as leg cellulitis, then confirmed with manual chart review. Each day the responsible physician (ID or EP) was documented. Other recorded variables of the chart review included: demographic factors, such as age and gender; decisions made by attending physicians, including daily antibiotic choice and dose, consults placed, imaging ordered, cultures of superficial wound swabs, discharge prescriptions and whether patients returned to the ED within one month of being discharged from the OPAT program. Patients that returned to OPAT within one month of discharge with an infection in the same leg were designated as insufficiently treated. If a patient returned outside of one month or with an infection present in the other leg this was considered a new infection and counted as a separate entry. Statistical analysis was done using R version 4.1.0 and excel version 16.79.1. Averages were compared using two tailed T-tests assuming unequal variance. The mean days in the OPAT program for our sample mostly followed a normal distribution with a coefficient of skewness less than 0.01.

## Results

Two hundred and nineteen patients met the inclusion criteria over the time-period. Of those, 81 were seen by an ID physician at least once and 138 patients were seen by EPs alone. Ages ranged from 18 to 97 years (median 55). Demographics were not significantly different between EP only and ID patients.

There was no statistically significant difference between mean number of days of treatment for patients seen by ID (3.88 days) or EPs only (3.4 days) (*p* value = 0.12). Patients seen by ID physicians at least once during OPAT were typically seen by EPs for several days before initially being seen by ID physicians. To mitigate this impact we calculated the mean number of days patients spent in OPAT after their first ID-supervised day and compared it to the average total number of days spent in OPAT for those not seen by ID physicians. This was statistically significant, with patients spending an average of 1.4 days in the program after being seen by ID physicians compared to 3.4 days in the program for those not seen by ID physicians (*p* < 0.0001).

Patients seen before November 2017 spent an average of 3.9 days in the OPAT program compared to a mean of 3.4 days for patients seen after increased ID physician involvement (*p* = 0.04).

We compared mean and median days in OPAT for patients categorized by the day when they first saw an ID physician. There was a clear trend that OPAT duration closely aligned with the day of initial assessment by ID physician (Table 1).

**Table 1:** Mean days in OPAT of patients based on the first day they were seen by ID physicians

First day seen by ID:	1	2	3	4	5	6	7	8
Mean days in OPAT	8.5	2.7	3.7	4.3	5.2	6.2	7	8.4
Median days in OPAT	1.5	2	3	4	5	6	7	8
Mean days in OPAT after ID consult	7.5	0.7	0.7	0.3	0.2	0.2	0	0.4
Number of patients seen by ID on each day (n)	2	22	12	8	5	5	2	5

Patients seen by ID physicians (35.3%) had cultures more often than patients seen by EPs (14.2%: *P* < 0.0001). ID patients grew methicillin-resistant *S. aureus (MRSA)*, group A *Streptococcus, Menterobacterales* and fungi more frequently. EP patients grew methicillin-susceptible *S. aureus* and *Streptococcus dysgalactiae* more frequently. ID physicians consulted more services than EPs (5.0% compared to 6.5%: *p* < 0.0001) including plastic surgery and vascular surgery (2.5% compared to 0.7%, and 1.3% compared to 0% respectively). While EPs were more likely to consult internal medicine and orthopedics (4.5% compared to 0%, and 1.5% compared to 1.2% respectively). Likewise, ID physicians ordered more imaging than EPs (26.5% compared to 18.7%: *p* < 0.0001). This result was consistent for computed tomography (CT), ultrasound (US) and X-ray when assessed independently. It was not feasible to assess differences in the rates of abnormal findings since the reports were narrative and the most common finding was soft tissue swelling consistent with cellulitis.

**Fig. 1:**
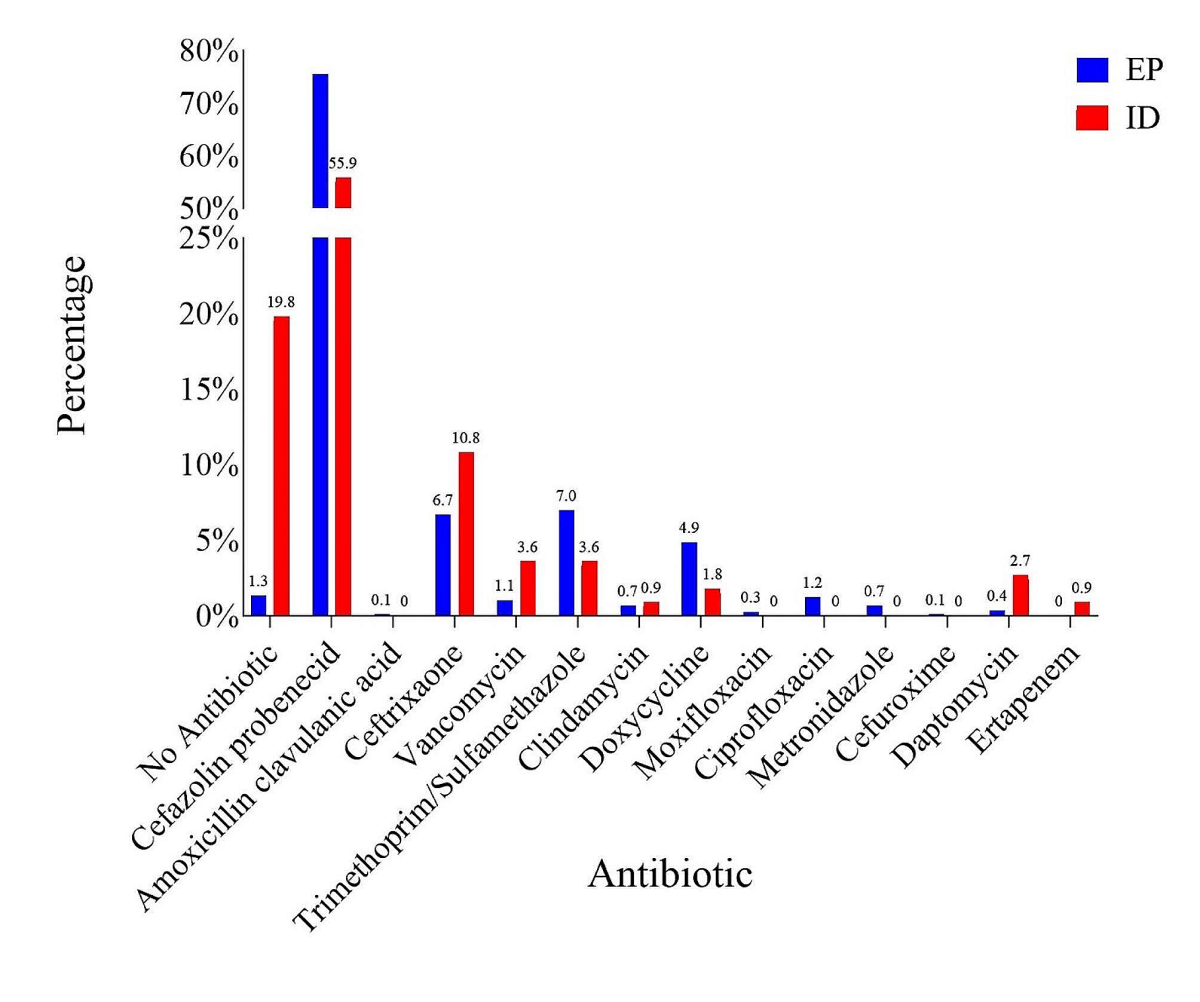
Antibiotics prescribed during OPAT by ID physicians and EPs. OPAT = outpatient parenteral antibiotic treatment; ID = infectious diseases; EP = emergency physician; TMP-SMX = trimethoprim/sulfamethoxazole

**Fig. 2:**
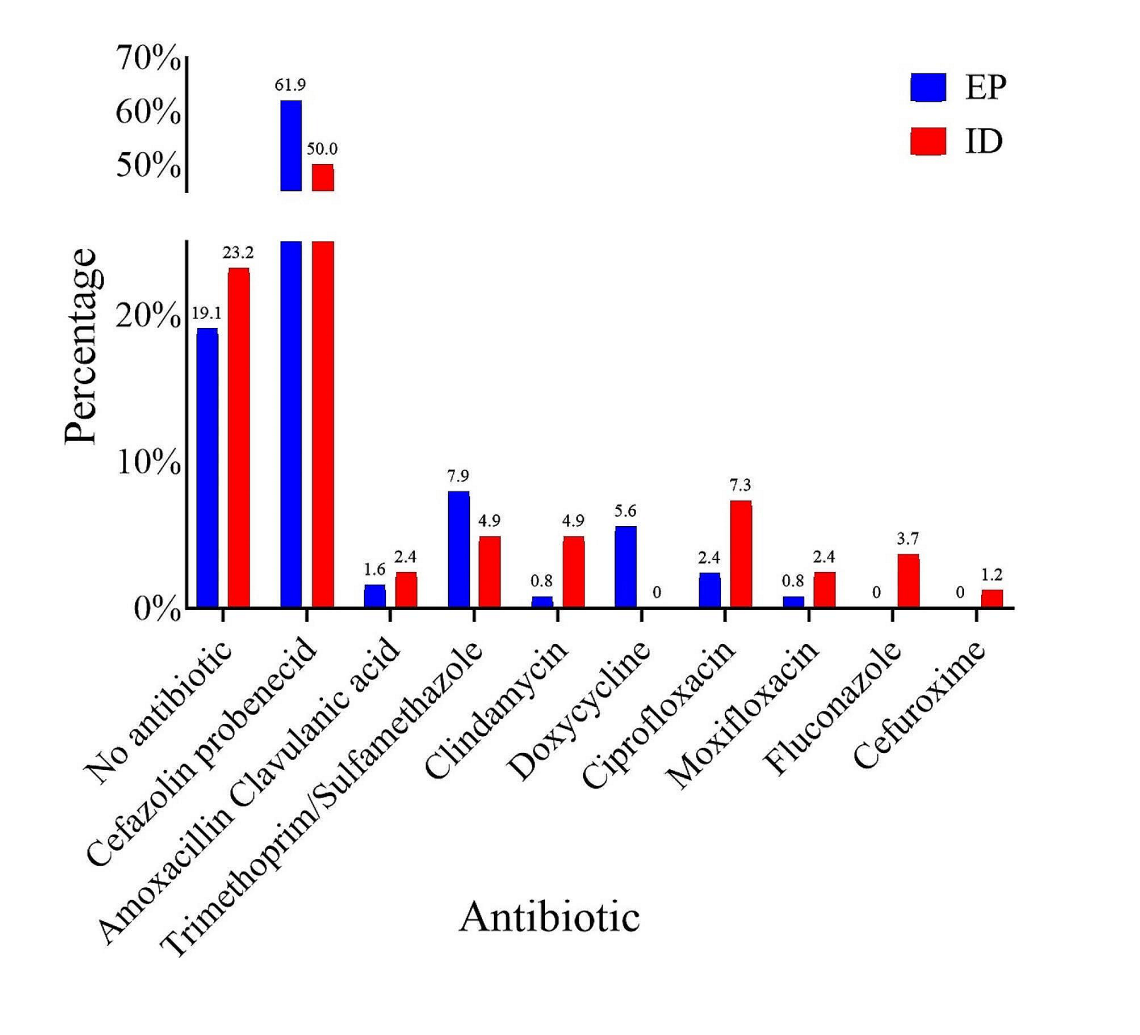
Antibiotics prescribed at discharge by ID physicians and EPs. ID = infectious diseases; EP = emergency physician; TMP-SMX = trimethoprim/sulfamethoxazole

Among antibiotics selected, EPs were more likely to prescribe the combination of daily IV cefazolin and oral probenecid, often combined with oral trimethoprim/sulfamethoxazole (TMP-SMX), or doxycycline. ID physicians were more likely to prescribe IV ceftriaxone, vancomycin, daptomycin, and ertapenem (Fig. 1), but were significantly more likely to stop IV antibiotics: 19.8% of the days they saw a patient compared to 1.3% of the days an EP saw a patient (*P* value < 0.00001). There were statistically significant differences in the antibiotic prescribed at discharge by ID physicians and EPs (Fig. 2): ID physicians prescribed no discharge antibiotic 21.6% of the time compared to 16.7% for EPs (*p* < 0.00001). Overall, ID physicians prescribed oral clindamycin, ciprofloxacin, moxifloxacin, amoxicillin/clavulanic acid, cefuroxime, and fluconazole more commonly.

The final diagnosis of patients seen by ID physicians varied more than those seen by EPs alone. Patients seen by only EPs had cellulitis as a final diagnosis 91.3% of the time compared to 81.5% for ID physicians. ID physicians diagnosed venous stasis, hematoma, edema, osteomyelitis, insect bites and gout more frequently than EPs. Only ID physicians diagnosed paronychia, vascular insufficiency, septic arthritis, and musculoskeletal pain. Only EPs diagnosed post-operative complications and allergic reactions.

Lastly, patients only seen by EP physicians were more likely to return with treatment failure compared to those seen by ID physicians (7.25% and 6.17% respectively: *p* < 0.0005).

## Discussion

Diagnosing cellulitis can be difficult as many diseases can masquerade as cellulitis: Deep vein thrombosis, contact dermatitis, gouty arthritis, and cutaneous manifestations of peripheral vasculopathy [4, 5]. A study by David et al. showed that 28% of cellulitis diagnoses made by EPs were determined to not be cellulitis when reassessed by dermatology or ID physicians [6]. While ID physicians are known to effectively run OPAT programmes: A multicentered ID-supervised OPAT study showed that 94% of patients were effectively treated [7], however no studies have compared OPAT programs supervised by ID physicians to those supervised by EPs. We predicted that ID specialist involvement would result in fewer days patients spent in the OPAT program, mitigating burden on the healthcare system and patients. Directly comparing outcomes of patients treated by EPs to those treated by ID physicians at the same hospital can quantify this comparative benefit. Further, assessment of ID physicians’ involvement in the VGH OPAT program has not been formally done.

When directly compared, there was no statistically significant difference in the number of days patients spent in the OPAT program when seen by ID physicians in comparison to those seen by EPs. However, this may undersell the value of ID contribution: ID physicians had limited availability, meaning ID physicians often became involved only with patients who were in the program after 5 days, selecting for more complicated cases that could not be discharged earlier. Also, some of the more obviously complicated patients, such as those with osteomyelitis, were referred to ID earlier, inflating the average number of days of treatment following ID consultation.

It was noted that patients in OPAT would frequently be discharged from the program after their first time being assessed by an ID physician but that this would often be after several days in the OPAT program being seen by EPs. This would inflate the number of days that patients seen by ID physicians spent in the program without reflecting ID physicians decisions. To mitigate this we calculated how many days patients spent in the OPAT program after their first assessment by an ID physician. We compared this to the total number of OPAT days for those seen by EPs alone, which showed that after being seen by an ID physician, patients spent a mean of 2 days less in the program compared to the number of days in OPAT for patients seen by EPs alone. It should be noted that this comparison wrongly disregards days of IV treatment patients received before being seen by ID physicians as negligible.

The mean number of days in the program and the day of initial ID physician’s assessment were closely aligned, except for day 1 (Table 1), which supports the premise that ID physician supervision decreases the number of days patients spend in the OPAT program. The exception seen on day one is an outlier with a sample size of one.

Comparison of cohorts, pre and post ID involvement, showed that patients spent an average of 0.5 days less in the OPAT program when ID was involved with a 14.9% reduction in treatment failure compared to EPs alone. There is the possibility that patients with recurrent infections presented at a different hospital, however this rate should not be different between ID physician and EP supervised patients.

We also found that ID physicians varied more in antibiotic prescriptions, final diagnosis and the organisms cultured. The antibiotic choices were more frequently narrow spectrum or oral antibiotic agents, presumably to target specific pathogens with less reliance on algorithmic treatment choices. For example, ertapenem was not prescribed by EPs, likely because any complex case requiring ertapenem would have prompted an ID physician referral.

ID physicians were more likely to discharge people without antibiotic prescriptions, including on the day of first assessment. This partly reflects the practice of ID physician assessment before medication administration, but also ID physician comfort in ruling out infection. In comparison, patients seen by EPs would commonly be given IV treatments while they waited to be assessed by an EP.

It’s possible that EPs may be more reluctance to question the diagnosis of a colleague. EP physicians were less likely to order additional tests such as cultures or imaging and requested fewer consults to other specialists. This could also be due to increased patient volume in the ED, algorithmic decision making or limitations in having tests reviewed, if ordered. It is also possible that tests were ordered less frequently if ID physicians became involved at the point that the need became apparent. Further, it should be noted that the increased cultures ID physicians ordered likely contributed to their use of a greater variety of antibiotics.

The higher frequency of consultation of internal medicine by EPs reflects the admission process in our hospital: admission requires an internal medicine consult. It is likely that patients failing initial outpatient treatment or with positive blood cultures were selected for hospital admission by the EPs in lieu of continued OPAT visits with subsequent ID physician assessment. The increased consultation of vascular and plastic surgery by ID physicians reflects the complex nature of ambulatory infections such as diabetic foot infections, which were likely to have been referred for ID physicians’ involvement and/or to require longer antibiotic courses.

A major limitation to our study was the inability to make a direct comparison between decisions made by EPs to ID physicians. We made several comparisons described above to identify substantial differences in management including comparing both by time period and by intervention group. However, the results will remain confounded. It is also important to note that the presence of ID physicians may have altered EP practices in the second time period, either through curb-side discussions or observing practice patterns. Future analysis can look at comparing more directly with a larger sample size of patients seen by ID physicians alone, which will hopefully be easier now that the OPAT program is run separate from the ED at VGH.

## Conclusion

The involvement of ID physicians was associated with 0.5 days fewer spent in the OPAT program without increasing the number of patients that returned to the ED with treatment failure. However multiple confounding variables complicate direct comparison. Since the conclusion of this study, the VGH OPAT program has been moved to a separate clinic from the ED and is now run entirely by ID physicians providing an opportunity for further comparisons with fewer confounders. An area for further study would be detailed comparisons and analysis of the increased variability of diagnosis and prescriptions that we observed. An increased sample size and more resources could elucidate implications and causes for these findings.

### Highlights

- Infectious disease involvement in OPAT decreases average days on IV antibiotics.
- Infectious disease physicians are less likely to prescribe antibiotics for OPAT patients.
- Infectious disease physicians use a greater variety of antibiotics in OPAT treatments.

### Electronic supplementary material

Below is the link to the electronic supplementary material.

Supplementary Material 1

Supplementary Material 2

## Data Availability

The datasets generated and/or analysed during the current study are not publicly available due confidentiality but are available from the corresponding author on reasonable request in de-identified form. The datasets supporting the conclusions of this article are submitted as additional files for peer review.
