# Supplementary material for: Quantitative analysis of the impact of infectious disease physicians on patients in the emergency department fast-track parenteral antibiotics program
Source: BMC Infect Dis. 2024 Jul 1;24:655. doi: 10.1186/s12879-024-09305-0 (PMC11218097; doi:10.1186/s12879-024-09305-0)
Supplement: Supplementary file 2 — Supplementary Material 2 [file 12879_2024_9305_MOESM2_ESM.docx]

Data guide for Quantitative Analysis of the Impact of Infectious Disease Physicians on Patients in the Emergency Department Fast-Track Parenteral Antibiotics Program manuscript

Legend: explaining what numbers mean for each category

Main data set: complete data including MRN, date, age, gender, day of ID consult, days on opat after ID consult, discharge disposition, return within one month, initial diagnosis, longest consecutive days in the opat program, total days in the program 1 month, final diagnosis discharge prescriptions, duration of prescription, cultured organisms, consults ordered, imaging ordered, allergies, AMA status and antibiotic choice for each day of treatment.

Full data set: all of the above as well as vitals for each day the patient was in OPAT

General data set: All data excluding data from individual days. MRN, date, age, gender, day of ID consult, days on opat after ID consult, discharge disposition, return within one month, initial diagnosis, longest consecutive days in the opat program, total days in the program 1 month, final diagnosis discharge prescriptions, duration of prescription, cultured organisms, consults ordered, imaging ordered, allergies, AMA status.

Demographic data:

Mean age, mean age by gender, gender distribution, mean days in regards to ID consult time, diagnoses, age distribution, consults, bacterial cultures, statistical analysis.

Research Questions:

1) Do patients seen by ID doctors spend less time in the OPAT program than those seen by EP physicians only?

No. Results are not statistically significant

| EP | 4.60 days |
| --- | --- |
| ID | 4.68 days |

2) Do patient seen by ID spend less days in the OPAT program following their initial ID assessment compared to the number of days spent in the OPAT program after an initial EP assessment for those patients not seen by ID?

Yes. Statistically significant

| EP | 3.43 days |  |
| --- | --- | --- |
| ID | 1.44 days |  |

3) Are patients seen by ID doctors more likely to have cultures taken than those seen by just EP doctors?

Yes. Statistically significant

|  | Percentage with cultures | |  |
| --- | --- | --- | --- |
| Not Seen By Infectious Diseases | 14.18% |  |  |
| Seen By Infectious Diseases | 35.29% |  |  |

4) Do the cultures from patients seen by ID physicians grow different organisms than those taken by EPs?

Yes, statistically significant

5) Do patients seen by ID doctors have CT’s, USs and Xrays than EP doctors

Yes, statistically significant

6) Do patients seen by ID doctors receive more imaging in general than EP doctors?

Yes, statistically significant

7) Do patients seen by ID doctors have more consults in general than those seen by only EP doctors?

Overall no. It seems to be that ID doctors ordered no IM consults. This is very unlikely so I think that there is a different process that the ID doctors use to request their IM consults considering they are themselves IM doctors.

8) Do patients seen by ID doctors receive more IM, Plastic, Vascular and Ortho consults than those seen by only EP doctors?

Patients seen by ID have more Vascular and plastic consults

9) Do ID doctors and EPs prescribe a home antibiotic with the same frequency?

No, ID are less likely to send patients home on antibiotics. Statistically significant.

10) Do ID doctors and EPs prescribe the same home antibiotics with the same frequency?

No, ID doctors prescribe certain antibiotics much more frequently, e.g. clinda, cipro and moxi

11) Do patients seen by ID doctors return within 1 month more frequently than those seen by EP doctors?

No, patients seen by EPs only had higher rates of returning. This was statistically significant.

12) Do ID and EP doctors differ in the final diagnosis for patients

Yes, ID doctors were less likely to diagnose just cellulitis and had much more varied diagnoses

13) Do ID doctors and EPs prescribe the same IV antibiotics with the same frequency?

No, ID doctors prescribe certain antibiotics much more frequently, e.g. ceftriaxone, daptomycin or no antibiotic

14) Do Opat patients spend less days in the program on average after the date of increased ID involvement in the program?

Yes, 3.4 days compared to 3.9 days.
